# The sequencing of the key genes and end products in the TLR4 signaling pathway from the kidney of *Rana dybowskii* exposed to *Aeromonas hydrophila*

**DOI:** 10.1515/biol-2022-0704

**Published:** 2023-09-16

**Authors:** Boju Wang, Jie Shao, Lili Qu, Qing Xu, Dong Zheng

**Affiliations:** College of Wildlife Resources, Northeast Forestry University, Harbin 150040, China

**Keywords:** kidney, long non-coding RNA, *Rana dybowskii*, single-molecule real-time sequencing, toll-like receptors

## Abstract

Infectious diseases caused by *Aeromonas hydrophila* (AH) have reduced the populations of *Rana dybowskii*). However, little is known about the immune response of *R. dybowskii* against AH infections. The toll-like receptor (TLR) signaling pathway has been identified as a critical component in innate immunity, responsible for identifying pathogen-associated molecular patterns in pathogens. Our study used the next-generation sequencing technique and single-molecule long-read sequencing to determine the structures of transcript isoforms and functions of genes in the kidneys of *R. dybowskii*, as well as identify and validate the related genes in the TLR4 signaling pathway. In total, 628,774 reads of inserts were identified, including 300,053 full-length non-chimeric reads and 233,592 non-full-length reads. Among the transcriptome sequences, 124 genes were identified as homologs of known genes in the TLR4 pathway especially inflammatory cytokines and receptors. Our findings shed light on the structures and functions of *R. dybowskii* genes exposed to AH and confirm the presence of both MyD88-dependent and independent pathways in *R. dybowskii*. Our work reveals how various functional proteins in amphibians at the initial stage of immune response are activated and complete their corresponding functions in a short time.

## 1 Introduction

*Rana dybowskii* is a significant amphibian species that is found primarily in northeast China. Some of its organs and products have high economic value and are used in traditional Chinese medicine [1,2]. Excessive hunting and infectious diseases caused by pathogens have resulted in a decrease in both wild and cultured populations of *R. dybowskii* [3–5]. The outbreak of *Aeromonas hydrophila*, among other pathogens, has contributed the most to the infectious diseases in *R. dybowskii* [6–8]. *A*. *hydrophila* has the potential to cause red leg disease in farmed frogs, resulting in massive economic losses [9–11]. However, effective methods for controlling the *A*. *hydrophila* outbreak have yet to be developed. Furthermore, susceptibility to *A*. *hydrophila* varies across populations and aquatic environments [12]. As a result, determining the immune response mechanisms of *R. dybowskii* against *A. hydrophila* is necessary and urgent.

Antimicrobial peptides have been shown to be important innate immunity-related effectors in providing resistance against microorganisms [9,13]. Toll-like receptor (TLR) signaling pathways regulate bactericide peptide levels [14]. Previous research has identified a number of TLRs [15,16]. TLRs are a type of pathogen recognition receptor that recognizes pathogen-associated molecular patterns (PAMPs) [17]. TLR genes play an important role in fungi-induced infection resistance because their homologs in the genome are capable of producing proinflammatory factors (type I interferon [IFN], tumor necrosis factor [TNF], and interleukin [IL]) in response to various pathogens [18,19]. Adult frogs and tadpoles both have frog TLRs. Tadpoles’ immunity is insufficient because they rely primarily on innate immunity (including TLR pathways) for resistance to microbial infections [7,20]. However, there is a scarcity of high-quality *R. dybowskii* transcriptomic and genomic data, which prevents a thorough examination of their immune system.

TLRs have many family members, but *A. hydrophila* is a Gram-negative bacteria with a unique identification substance called lipopolysaccharide (LPS). LPS, as an important PAMP, can and only causes the activation of the TLR4 signal pathway. Based on previous research on the TLR4 signaling pathway, we believe that TLR4 should have at least two pathways to activate the expression of end products in frogs in order to achieve the immune effect.

Because of emerging next-generation sequencing (NGS) and single-molecule real-time (SMRT) sequencing technologies, transcriptomic analysis and genomic sequencing methods have become feasible in recent years [21,22]. Notably, SMRT sequencing greatly simplifies transcriptomic and genomic research by providing necessary structural and functional data for genes in a variety of organisms [23]. Transcriptomic analysis, on the other hand, has been significantly altered by high-throughput NGS, which can now be used in nearly all fields [24]. Overall, the combination of SMRT and NGS has greatly aided comprehensive transcriptomic analysis [25].

This study used SMRT sequencing to identify *R. dybowskii*’s full-length transcriptome within renal tissues after injecting it intraperitoneally with *A. hydrophila*. Following that, we used NGS to obtain short reads of corrected transcript isoforms. Later, we annotated the transcript isoforms of *R. dybowskii*, which helped us understand the complex mechanisms of *R. dybowskii*’s immune response. To determine immune patterns in *R. dybowskii* exposed to *A. hydrophila*, the transcriptome expressions of inflammatory cytokines and receptors were examined. As a result, our work generated valuable genetic resources and transcriptome data to aid future research on *R. dybowskii* and investigate the potential immune response mechanisms against *A. hydrophila*.

Although the research method we used is a widely used and mature research method in higher animals, such as mammals, our research is the first for amphibians. So our work can help humans have a more comprehensive and systematic understanding of how amphibians respond to bacterial infections. Our work also provides basic data for researchers who want to understand the immune function of amphibians, and we have organized and summarized this part of the data for other researchers to refer to.

At the same time, the focus of our laboratory’s research has always been the immune response of amphibians, not only because amphibians, especially the *R. dybowskii*, are important economic animals, but also because amphibians are a kind of organisms that can be called “environmental monitoring organisms.” Because of their special physiological structure and lifestyle, amphibians are more vulnerable to microbial infection. In response to this situation, amphibians have developed unique and highly effective immune response systems. We believe that through in-depth research on the immune system of amphibians, we can not only deepen our understanding of amphibians, but also hope to provide new and more effective antibacterial products.

## 2 Materials and methods

### 2.1 Sample collection and preparation

Thirty wild, healthy male frogs were captured in the Yichun area of Heilongjiang Province. All animals used in experiments were treated ethically in accordance with Chinese laws and regulations governing Science and Technology applications. The frogs were randomly assigned to either the control or experimental groups (*n* = 15 each). The experimental frogs were given an intraperitoneal injection of 1 mL *A. hydrophila* (10^7^ CFU/mL) (provided by the Institute of Microbiology, Chinese Academy of Sciences) that was originally cultivated and preserved at Northeast Forestry University’s Laboratory of Animal Physiology. Simultaneously, the control group received an intraperitoneal injection of 1 mL of LB liquid medium. The frogs were kept alive for 2 h before being euthanized by double marrow destruction to collect kidney tissue samples, which were then stored at –80°C until further use.


**Ethical approval:** The research related to animal use has been complied with all the relevant national regulations and institutional policies for the care and use of animals.

### 2.2 RNA preparation and complementary DNA (cDNA) library construction for the transcriptome analysis

To avoid errors in data analysis, we processed each group’s kidney samples separately and labeled them precisely, and TRIzol® Reagent (Ambion, USA) was used to extract total RNAs according to the specific instructions. To assess the integrity, quality, and quantity of the RNA, the Agilent Bioanalyzer 2100 system (Agilent Technologies, Palo Alto, CA, USA) and the Nanodrop method (NanoDrop Technologies, Wilmington, DE, USA) were used. The quantified RNA samples were then used to build cDNA libraries. Following purification, the mRNA was enriched in the combined total RNA using Oligo (dT) beads (Thermo Fisher Scientific, Waltham, USA). The mRNA was then fragmented into small fragments in the fragmentation buffer at high temperatures using divalent cations. The first strand of cDNA was then synthesized using a random hexamer primer and short fragments as templates. The second strand of cDNA was synthesized using a buffer containing DNA polymerase I, Ribonuclease H, and deoxyribonucleotide triphosphates, followed by cDNA purification using AMPure XP beads (Beckman, Brea, CA, USA). The double-stranded cDNAs with end-repair and poly(A)-tail were then ligated into the sequencing adapters. The size of the cDNA fragments was then selected using the AMPure XP beads once more, followed by enrichment via PCR. The qualifying libraries were sequenced on an Illumina HiSeq instrument (Illumina Inc., San Diego, CA, USA), and the raw sequence reads were deposited in the NCBI database.

### 2.3 Quality filtering and error correction in the PacBio long reads

Following amplification, we removed sequences of <50 bp or with a quality score of <0.75. Following that, we extracted the reads of inserts (ROIs) in those subreads by filtering and dividing them into non-full-length or full-length reads. Following that, we obtained clean reads by removing the low-quality reads and adapters. These clean reads were then used to correct the low-quality full-length transcript isoforms with the Proofread software. After correction, the de-reductance of low- and high-quality transcript isoforms was verified in order to obtain non-redundant ones.

### 2.4 Analysis of coding sequences (CDS)

Each open reading frame (ORF) was designated as a candidate CDS. Later, we classified the estimated CDS types as internal (no stop and start codons were estimated), complete (both stop and start codons were estimated), and 3′ end (3′-partial, only stop codon was estimated) or 5′ end (5′-partial, only start codon was estimated).

### 2.5 Functional annotation

We compared the transcriptome data from different treatment methods (experimental group and control group) at the same time to find the differentially expressed genes (DEGs). These DEGs were aligned with non-redundant protein sequences (NR), non-redundant nucleic acid sequences (NT), SwissProt, Kyoto encyclopedia of genes and genomes (KEGG), euKaryotic ortholog groups (KOG), InterPro, and gene ontology (GO), and the intersection was checked with Blast, Blast2GO, and InterProScan5.

### 2.6 Experimental validation of DEGs and the end products of TLR4 signal pathway

We used β-actin as an internal reference gene, after that, we designed merger primers for three end products, namely IFN, TNF, and IL. In order to investigate the types of end products that depend on two pathways in the TLR4 signaling pathway and the initiation time of the two dependent pathways, we also selected inhibitors of TLR4, MyD88, and TRIF molecules to join the experimental work. Frogs were infected with 1 mL of AH bacterial solution with the concentration of 10^7^ CFU/mL, then we executed the frog and collected the kidney from the frogs at six different time points after infection: 2, 4, 8, 12, 24, and 48 h. Subsequently, qRT-PCR detection was performed, and the experimental results were organized and produced into a time-expression histogram.

## 3 Results

### 3.1 General properties of the single-molecule long-reads

**Figure 1:**
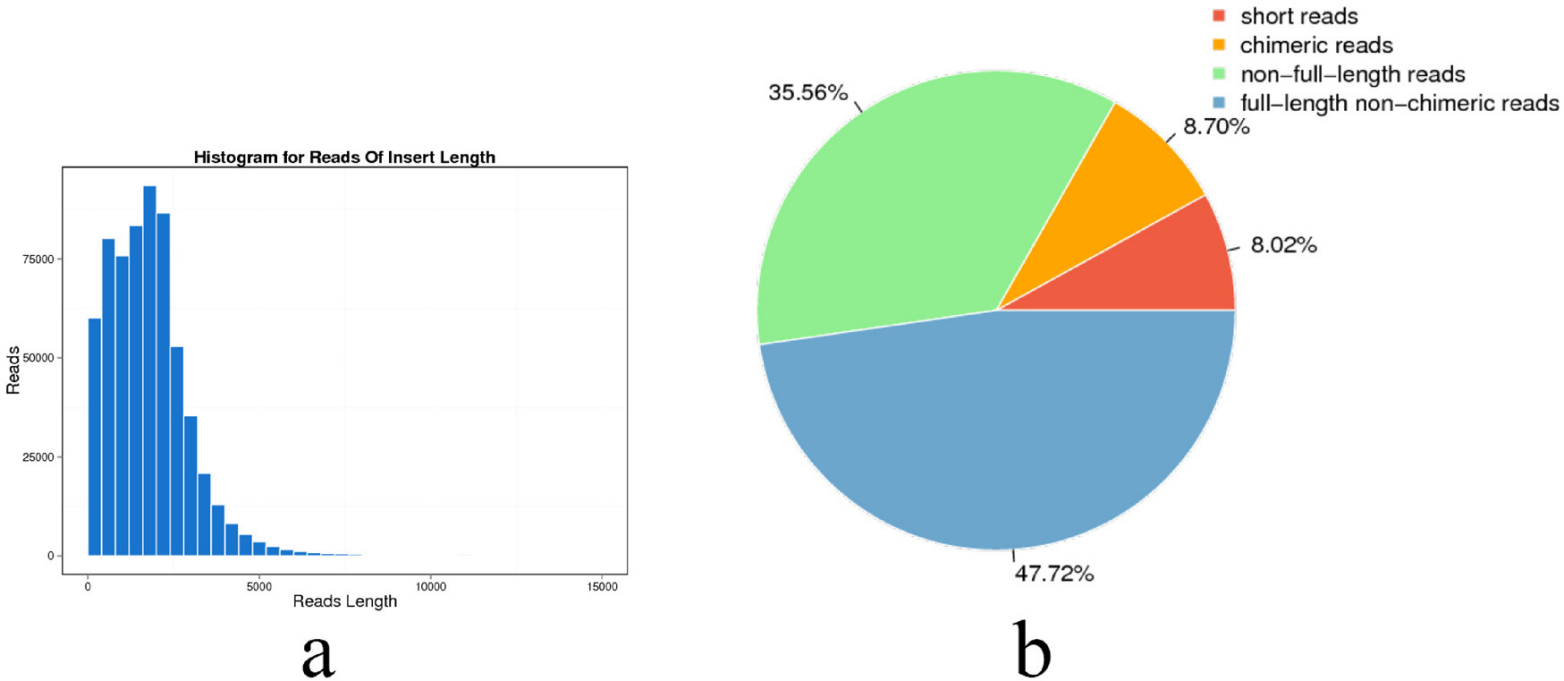
Histogram for the length of the ROI (a) and the summary of classification of the reads (b).

We performed an Illumina-based transcriptomic HTS analysis on transcriptomes of combined cDNA obtained from renal tissues to determine gene expression levels in *A. hydrophila*-exposed *R. dybowskii*. A total of 628,774 ROIs were generated, with the insert’s mean read length of 1,787 bp (Table 1 and Figure 1a). Of these 47.72% of ROIs (300,053) were full-length non-chimeric reads, while 35.56% of ROIs (233,592) were non-full-length reads (Figure 1b).

**Table 1:** Statistics of the ROI in *R. dybowskii*

cDNA size	Total
ROI	628,774
Five primer reads	445,002
Three primer reads	461,624
Poly(A) reads	437,951
Mean read length of the insert	1,787

### 3.2 Cluster and error correction of the long-reads

Full-length non-chimeric reads were clustered into a consistent sequence, and consensus isoforms were found in all clusters. The quiver program was used in conjunction with the non-full-length sequences to correct the consensus sequences for each cluster, resulting in high-quality transcripts with an accuracy rate of >99%. Finally, 142,128 transcript isoforms of high quality were obtained. At the same time, 161,684,714 clean reads were used to correct the low-quality consensus isoforms (Table 2). After removing the unnecessary transcripts, 116,812 transcriptional sequences were obtained, which could all be used in subsequent structural and functional analyses.

**Table 2:** Summary of the clustering information of full-length non-chimeric reads in *R. dybowskii*

Cluster type	Total isoforms	Total base (bp)	Mean quality	Mean isoform length (bp)	Mean full length coverage	Mean non-full length coverage
High quality	142,128	235,744,818	0.9987	1,659	1.39	18.94
Low quiver	96,298	161,684,714	0.4721	1,679	1.06	8.54

### 3.3 Predictions of CDSs

**Figure 2:**
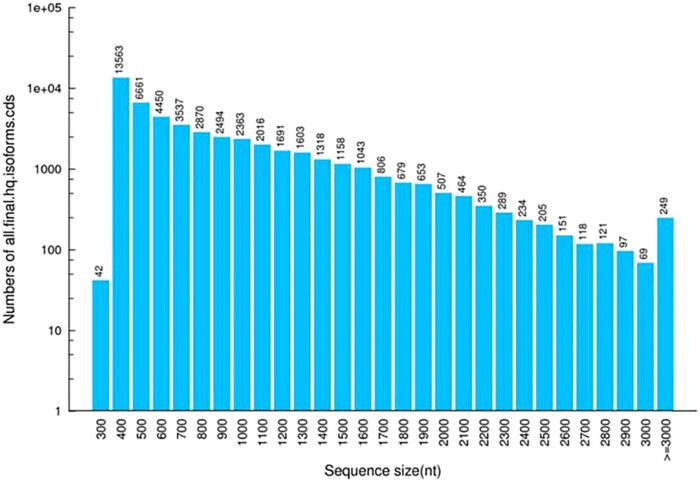
Number and length distribution of proteins encoded by the coding sequence regions in *R. dybowskii*.

Figure 2 represents length and quantity distributions of CDS region-encoded protein, where 49,801 ORFs were estimated with a total length of 40,476,447.

### 3.4 Functional annotation of the transcripts

**Figure 3:**
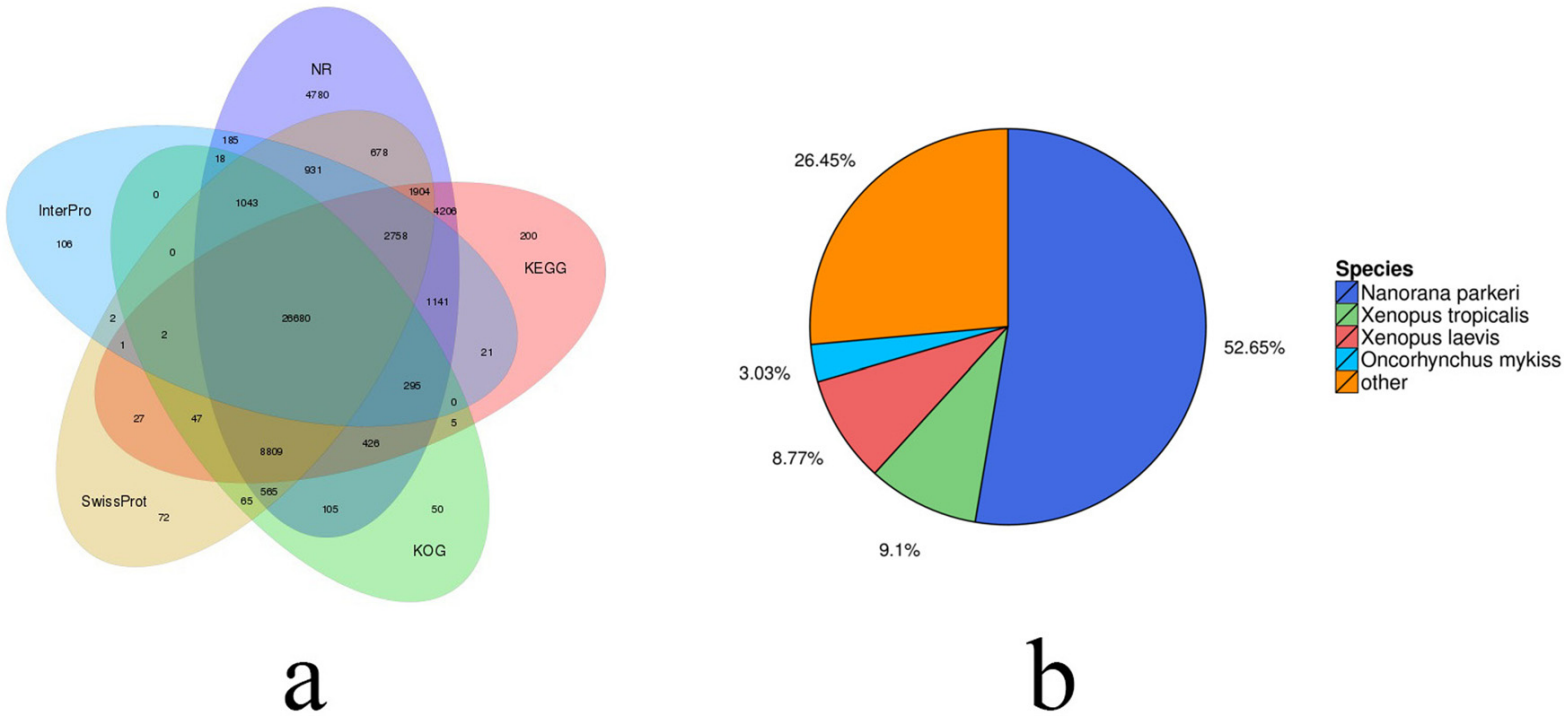
Numbers of annotated unigenes in five public databases (a) and the species most tightly associated with *R. dybowskii* obtained from the NR database (b).

To annotate the transcript functions, eight databases (NR, NT, SwissProt, KEGG, KOG, InterPro, GO, and Intersection) were used. Of the 116,812 transcripts, 81,744 (69.98%) were matched with at least one of the above databases, while 54,524 (46.68%), 71,766 (61.44%), 43,584 (37.31%), 46,522 (39.83%), 38,110 (32.63%), 33,183 (28.41%), 24,105 (20.64%), and 15,667 (13.41%) transcripts were not. However, we did not match the remaining ones, which could be *R. dybowskii*’s new transcripts. The Venn diagram depicts the total number of annotations in five public databases (Figure 3a), with 26,680 genes (22.84%) annotated in all databases.

Later, we compared the transcripts from *R. dybowskii* to those from other species found in the NR database. We discovered that the function of *R. dybowskii* genes can be annotated by *Nanorana parkeri*, *Xenopus tropicalis*, Xenopus *laevis*, and *Oncorhynchus mykiss*. The genes annotated through *N. parkeri* accounted for the highest proportion, 52.65%, of the genes labeled at least once. The annotated scale was 26.45% with the other species, and *X. tropicalis* (9.10%) and *X. laevis* (8.77%) belonged to the same genus (Figure 3b).

**Figure 4:**
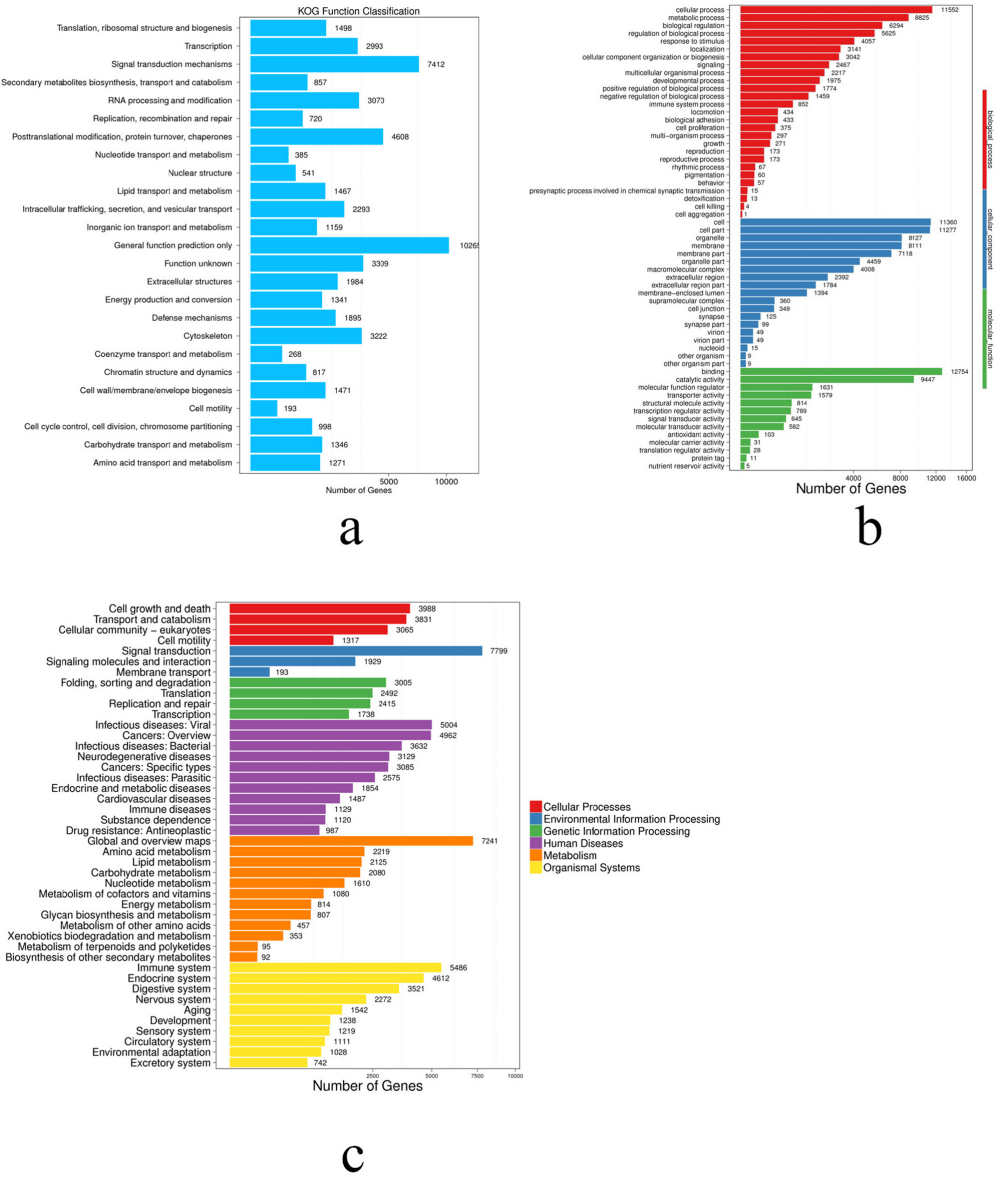
Functional classifications of annotated transcripts from *R. dybowskii*: (a) KOG function classification, (b) KEGG function classification, and (c) GO function classification.

Using the KOG database, we annotated 38,110 transcripts with complete functional data and classified them into 25 categories (Figure 4a). We assigned many transcript isoforms to only general functional estimation (26.94%), while others were assigned to signal transduction mechanism (19.45%).

We also used the KEGG database to annotate 101,400 transcripts in *R. dybowskii* into 126 metabolism-related pathways (Figure 4b), and discovered that the most significantly annotated pathways involved human diseases (28,964; 28.56%), metabolism (17,893; 17.65%), and organismal systems (22,771; 22.46%). Furthermore, the functional annotation revealed 7,799 signal transduction and 7,472 global and overview maps (Figure 6), with the KEGG database revealing 5,484 immune systems.

The GO terms were classified into three biological groups: biological process (BP, 28,419; 19.58%), cellular component (CC, 55,653; 38.34%), and molecular function (MF, 61,094; 43.09%) (Figure 4c). Cellular process (40.65%) was the most dominant biological process category, followed by metabolic process (31.05%), biological regulation (22.15%), and biological process regulation (22.15%) (19.79%). Transcript isoforms had a stronger relationship to cell (20.41%), cell part (20.26%), organelle (14.60%), and membrane in the CC category (14.57%). The majority of transcript isoforms in the MF category were annotated to binding (20.88%) and catalytic activity (15.46%).

### 3.5 Gene identification in the TLR4 pathway and inflammatory cytokines and receptors of other species

**Figure 5:**
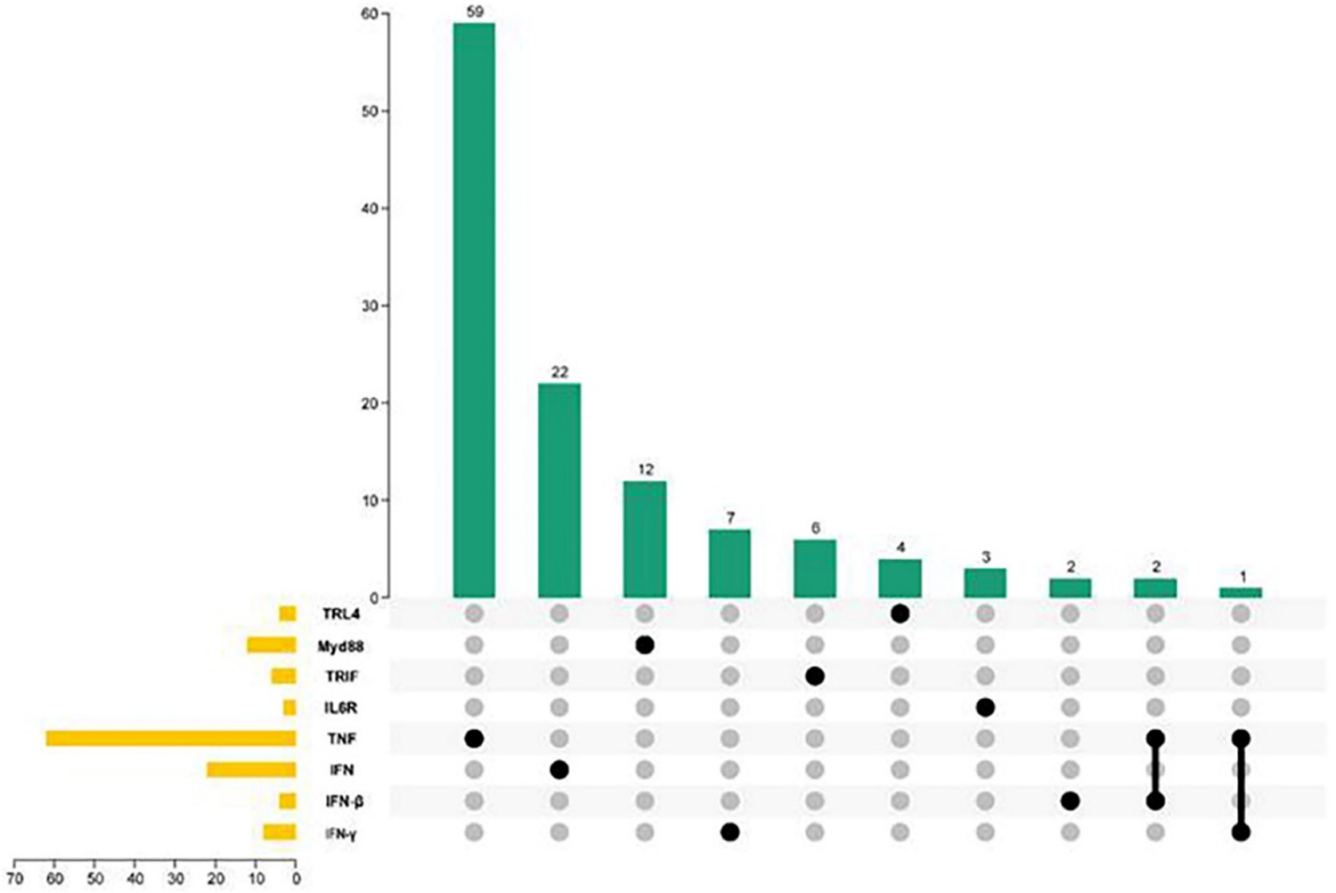
Gene identification in the TLR4 pathway andinflammatory cytokines and receptors of other species.

To better understand the mechanisms underlying the *A. hydrophila*-exposed *R. dybowskii*, 124 genes from other species were classified as having changed transcriptome expression after AH infection. There are 59 isoform gene fragments that belong to TNF and the same superfamily, 22 isoform gene fragments that belong to IFN, 2 isoform gene fragments that belong to IFN, 7 isoform gene fragments that belong to IFNγ, 3 isoform gene fragments that belong to IL6R, 4 isoform gene fragments that belong to TLR4, 12 isoform gene fragments that belong to MyD88, and 6 isoform gene fragments that belong to same type. At the same time, we discovered that three gene fragments could not be fully classified. Two gene fragments may exist in the IFN, ifn-b, and TNF superfamilies, and one gene fragment may exist in the TNF and IFN superfamilies. We classified the aforementioned genes and obtained the following results: 4 TLR4 genes, 12 Myd88 genes, 6 TRIF genes, 3 IL6R genes, 62 TNF genes, and 34 IFN genes (22 IFN, 4 IFNβ, and 8 IFNγ) were identified from homologs of known genes in TLR4 pathways and inflammatory cytokines and receptors of other species (Figure 5). *Isoform 91521* was identified as both TNF and IFNγ, among these genes, while *Isoform 71039* and *Isoform 91481* were identified as both TNF and IFNβ.

### 3.6 Transcriptome expression of inflammatory cytokines and receptors in *R. dybowskii* exposed to *A. hydrophila*

**Figure 6:**
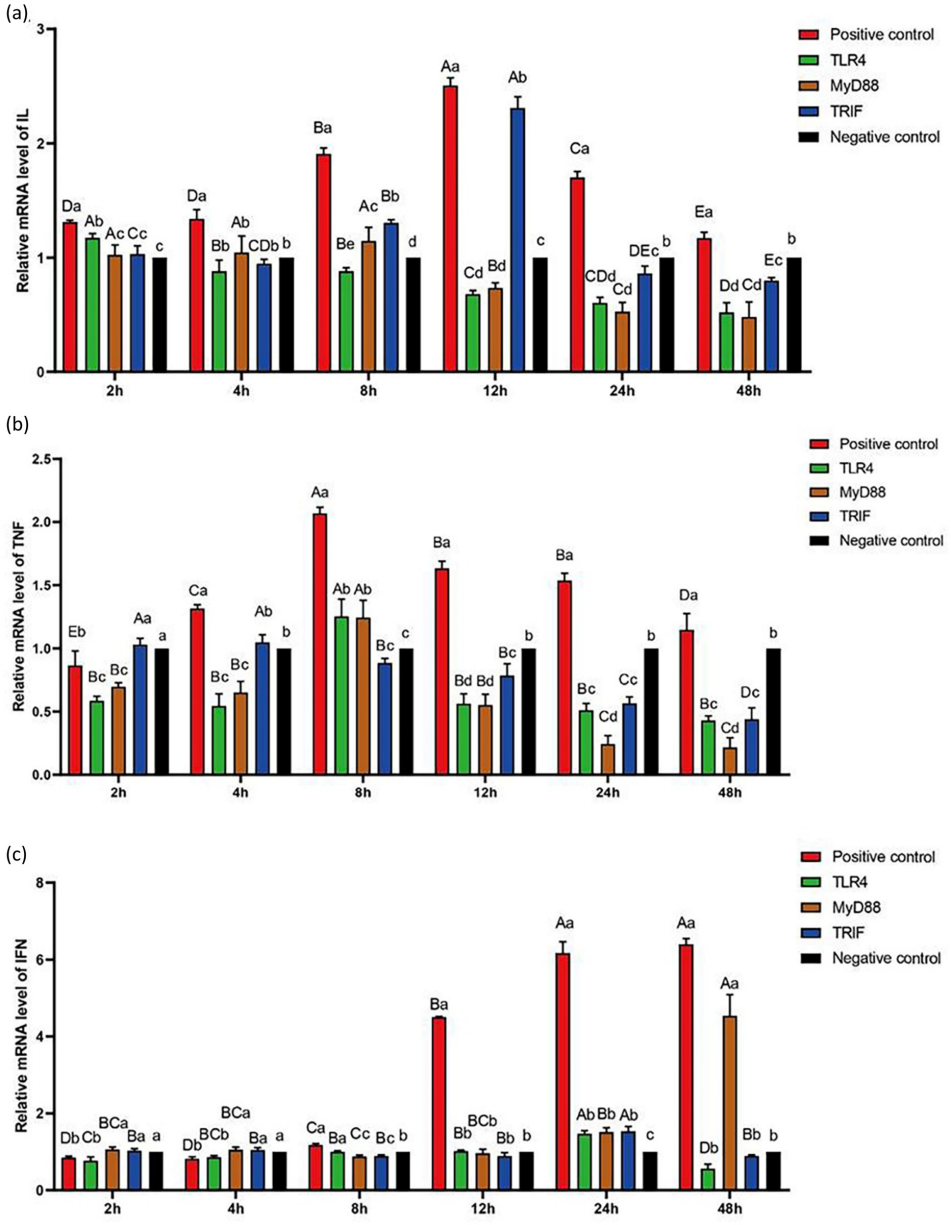
Relative expression levels of IL (a), TNF (b), and IFN (c) at different stimulation times of 2, 4, 8, 12, 24, and 48 h.

TLR could recognize different pathogen-related molecular patterns and its products could activate other immune-related pathways in *R. dybowskii* exposed to *A. hydrophila.* The three end products, IL, TNF, and IFN, were detected at 2, 4, 8, 12, 24, and 48 h after stimulation (Figure 6).

#### 3.6.1 Expression of IL

The response values for IL expression increased after stimulation. After 12 h, the values peaked and then began to fall. After adding inhibitors, the response value of IL expression decreased significantly. After the addition of TLR4 inhibitors, the response value of IL expression decreased over time, reaching a significantly lower value than the negative control after 8 h. Similarly, after adding a MyD88 inhibitor, the response value of IL expression decreased over time, reaching a significantly lower value than the negative control after 12 h. However, after the TRIF inhibitor was added, the response value for IL expression increased and peaked after 12 h, before declining. After 24 h, the response values were significantly lower than the negative control.

#### 3.6.2 Expression of TNF

After stimulation, the response value of TNF expression increased, peaked after 8 h, and then declined. After the addition of inhibitors, the response value of TNF expression was significantly reduced. After MyD88 and TLR4 inhibitors were added, the response value of TNF expression increased, peaked after 8 h, and then declined, showing a significantly higher value than the negative control at 8 h. However, after the addition of the TRIF inhibitor, the response value of TNF expression decreased over time, reaching a significantly lower value than the negative control after 12 h.

#### 3.6.3 Expression of IFN

The IFN expression response value increased with time after stimulation, reaching a significantly higher value than the negative control after 8 h. After the TLR4 inhibitor was added, the response value for IFN expression increased and peaked at 24 h, then declined, showing a significantly higher value than the negative control at 24 h. After the MyD88 inhibitor was added, the response value for IFN expression increased over time, reaching a significantly higher value than the negative control after 24 h. However, after the addition of the TRIF inhibitor, the response value for IFN expression increased and peaked at 24 h before declining. At 24 h, the response values were significantly higher than the negative control.

## 4 Discussion

Transcriptome sequencing is a powerful tool for determining the relative importance of expressed genes in specific materials at different times or under different conditions. It can be used to learn more about cell processes like growth, development, metabolism, and immune disorders [26]. To investigate the genes involved in *A. hydrophila*-exposed *R. dybowskii*, we used NGS and SMRT sequencing to perform full-length, high-quality transcript isoform sequencing. As a result, we were able to gain a better understanding of the mechanism underlying the actions of bacterial-infected *R. dybowskii*. We obtained 628,774 ROIs in total, including 300,053 non-chimeric full-length reads. Meanwhile, we discovered 142,128 consensus isoforms, including 116,812 high-quality transcript isoforms, after performing inference of CRISPR edits analyses on non-chimeric full-length reads. Following that, we used the Illumina platform to sequence the reads, corrected the errors, and used CD-HIT software to remove the unnecessary transcripts, yielding 116,812 necessary transcript isoforms. The high-quality SMRT data were also validated using integrative sequencing analysis. To begin, the overall mean length was determined to be 1,787 bp, indicating that ROIs of sufficient length represented full-length transcripts [27]. Second, low-quality SMRT reads were corrected using Illumina data to reduce SMRT sequencing errors [28]. Therefore, the resulting integrative *R. dybowskii* transcriptome database could shed more light on *R. dybowskii* gene composition and functional activities.

According to the NR analyses, *R. dybowskii* is highly homogeneous to *N. parkeri* and can be used as a reference for research on a variety of topics. Fortunately, extensive research on frogs and their immune response mechanisms have been conducted. The resulting unigene sequences were found in a variety of databases, including KOG (26.94% of the transcript isoforms were assigned to only general function prediction, while 19.45% were assigned to signal transduction mechanism), KEGG (28.56% were found in human diseases, 17.65% in metabolism, and 22.46% in organismal systems), and GO (19.58% were found in biological process, 38.34% in the cellular component, and 43.09% in molecular function). Specifically, 5,484 immune systems were discovered using the KEGG database. Such immune pathways, particularly the TLR4 pathway, were suggested to play critical roles in *R. dybowskii* exposed to *A. hydrophila.*

We identified 4 TLR4 genes, 12 Myd88 genes, 6 TRIF genes, 3 IL6R genes, 62 TNF genes, and 34 IFN genes (22 IFN, 4 IFNβ, and 8 IFNγ) in the kidney transcriptome of *R. dybowskii* exposed to *A. hydrophila* from homologs of known genes in the TLR4 pathway and inflammatory cytokines and receptors of other species. Common proinflammatory cytokines such as IL, TNF, and IFN are increased in *R. dybowskii* after exposure to *A. hydrophila*. TLR4 can induce the expression of IL, TNF, and IFN to amplify inflammatory signals, thereby initiating the inflammatory cascade. TLR4 inhibitors, on the other hand, can reduce IL, TNF, and IFN expression. After adding the TLR4 inhibitor, the response value for IL expression decreases over time, whereas the response value for TNF and IFN expression increases and peaks at 8 and 24 h, respectively, before declining. Furthermore, after 8 h, the expressions of IL and TNF were significantly lower than the negative control, while IFN expression was higher than the negative control at 24 h. As a result, we hypothesized that this species had both MyD88-dependent and independent pathways. The MyD88-dependent pathway was activated relatively early, which may have aided in the activation of the independent pathway.

We simulated the structure of the protein and discovered its functional site by constructing MyD88 and TRIF gene sequences and analyzing protein structure. Simultaneously, we discovered inhibitors of corresponding proteins in mammals and fish. Previous research indicates that these inhibitors can be used normally in frog animal models. We determine the dependency between the end products and the signal pathway by suppressing some key nodes in the signal pathway.

## 5 Conclusions

We used mature technologies in mammals to explore the immune system of amphibians that have not yet been studied, deepening people’s overall understanding of the immune system of vertebrates. Using a combination of SMRT sequencing and NGS technology, high-quality full-length non-chimeric transcripts, ORFs, SSRs, and lncRNAs were detected in *R. dybowskii* exposed to *A. hydrophila*. *R. dybowskii* is found to be very similar to *N. parkeri.* Based on the sequencing results, we spliced and constructed the structure and signal transduction sequence of each molecule in the amphibian TLR4 signaling pathway, and obtained the important conclusion that the immune system in vertebrates is relatively conservative. To shed more light on the mechanisms underlying the immunity of *A. hydrophila*-exposed *R.* dybowskii, we identified 124 genes from transcriptome sequences in known gene homologs within the TLR4 pathway as well as inflammatory cytokines and receptors from other species. In *R. dybowskii*, both MyD88-dependent and independent pathways were discovered. At the same time, we obtained the relationship between the end products of different dependent pathways in the frog TLR4 signaling pathway by using inhibitors. We recognized through the results of inhibitor experiments that the MyD88 dependent pathway is activated in the early stages of bacterial invasion, and found that the My88 dependent pathway promotes the activation of the MyD88 independent pathway. Our findings also contribute to a better understanding of the gene structure and functional activities in *R. dybowskii* exposed to *A. hydrophila*. Furthermore, it confirms the involvement of both the MyD88-dependent and MyD88-independent pathways in *R. dybowskii*.
